# Supplementary material for: Interpretable embeddings of speech explain and enhance the brain encoding performance of audio models
Source: ArXiv. 2026 Oct 20:arXiv:2507.16080v3. Preprint. [Version 3] (PMC13738929)
Supplement: Supplementary file 1 [file NIHPP2507.16080v3-supplement-1.pdf]

Supporting information

S1 Fig. Unique neural variance by each interpretable feature. Unique neural variance R^2 accounted for by each feature with respect to the rest of the interpretable features. The language processing hierarchy can be clearly observed in the anatomical distributions.

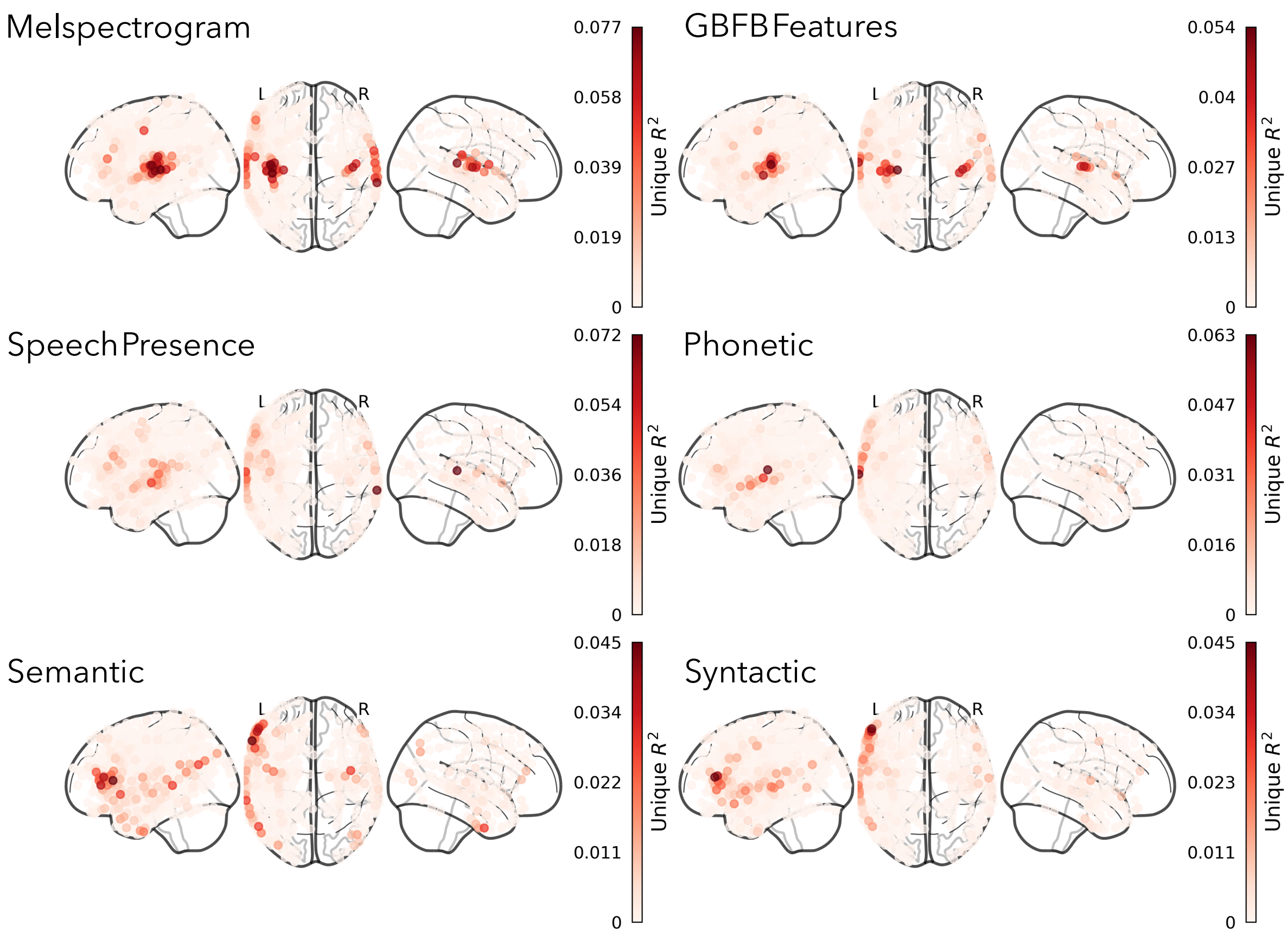

S2 Fig. Histogram of word count in a 0.5-second window. The histogram of number of words in a 0.5-second window used to obtain linguistic features.

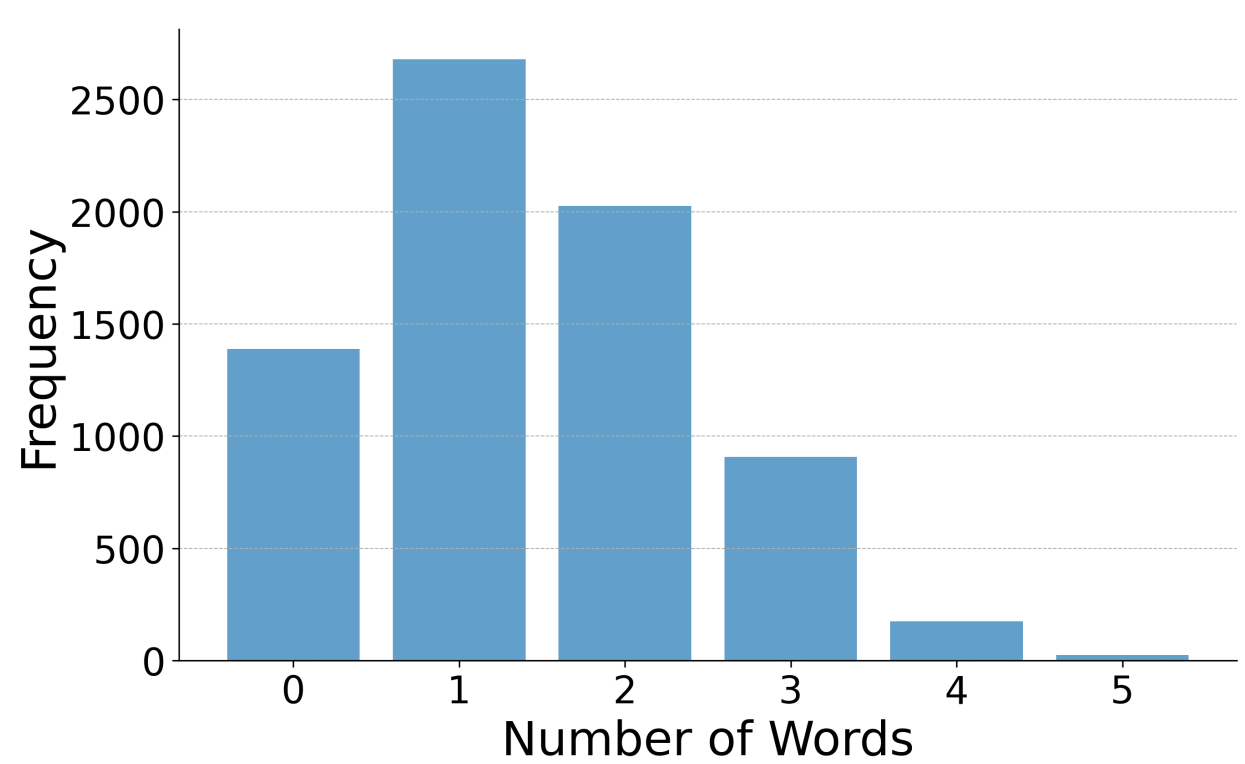

S1 Table. List of all questions in question-answering features.

Question
1. Does the sentence describe a personal reflection or thought?
2. Does the sentence contain a proper noun?
3. Does the sentence describe a physical action?
4. Does the sentence describe a personal or social interaction that leads to a change or revelation?
5. Does the sentence involve the mention of a specific object or item?
6. Does the sentence involve a description of physical environment or setting?
7. Does the sentence describe a relationship between people?
8. Does the sentence mention a specific location?
9. Is time mentioned in the input?
10. Is the sentence abstract rather than concrete?
11. Does the sentence express the narrator's opinion or judgment about an event or character?
12. Is the input related to a specific industry or profession?
13. Does the sentence include dialogue?
14. Does the sentence describe a visual experience or scene?
15. Does the input involve planning or organizing?
16. Does the sentence involve spatial reasoning?
17. Does the sentence involve an expression of personal values or beliefs?
18. Does the sentence contain a negation?
19. Does the sentence describe a sensory experience?
20. Does the sentence include technical or specialized terminology?
21. Does the input contain a number?
22. Does the sentence contain a cultural reference?
23. Does the text describe a mode of communication?
24. Does the input include a comparison or metaphor?
25. Does the sentence express a sense of belonging or connection to a place or community?
26. Does the sentence describe a specific sensation or feeling?
27. Does the text include a planning or decision-making process?
28. Does the sentence include a personal anecdote or story?
29. Does the sentence involve a discussion about personal or social values?
30. Does the text describe a journey?
31. Does the input contain a measurement?
32. Does the sentence describe a physical sensation?
33. Does the sentence include a direct speech quotation?
34. Is the sentence reflective, involving self-analysis or introspection?
35. Does the input describe a specific texture or sensation?
36. Is the input about a discovery or realization?
37. Does the sentence include an account of a miscommunication or misunderstanding?
38. Does the sentence include a specific sound or auditory description?
39. Does the sentence use a unique or unusual word?
40. Does the sentence describe a change in a physical or emotional state?
41. Does the sentence describe a moment of relief or resolution of tension?
42. Does the sentence include a conditional clause?
43. Does the sentence reference a specific time or date?
44. Is the sentence conveying the narrator's physical movement or action in detail?
45. Is there mention of a city, country, or geographic feature?
46. Does the sentence involve an unexpected incident or accident?
47. Does the sentence involve a recount of a social or community event?
48. Does the sentence express a philosophical or existential query or observation?
49. Does the story involve a personal project or creation?
50. Is the sentence emotionally positive?
51. Does the sentence describe an activity related to daily life or routine?
52. Does the text include a reference to a past era or time period?
53. Does the input discuss a societal issue or social justice topic?
54. Does the sentence convey a decision or choice made by the narrator?
55. Does the sentence convey a sense of urgency or haste?
56. Is the sentence providing an explanation or rationale?

S2 Table. Summary of the speech foundation models used in this study.

Model	# Layers	# Dimensions	# Parameters	Training Objective
HuBERT X-large	48	1280	964M	Masked prediction of *quantised* latent units from iterative k-means clustering of MFCCs
WavLM Large	24	1024	317M	Same masked-unit objective as HuBERT + utterance-mixing and denoising augmentation
Whisper Large	32	1280	638M	Supervised multilingual encoder–decoder ASR and speech-to-text translation

S3 Table. Control for lexical predictability. Six word-level predictors of lexical predictability were added to the non-semantic control set: unigram log-frequency, bigram and trigram log-probability, skip-gram log-probability $\log P(w_t \mid w_{t-2})$, per-word GPT-2 surprisal, and the predictive entropy of the next-token distribution. The n-gram probabilities are from an interpolated trigram model counted over the Brown and Reuters corpora (2.5 M tokens); the GPT-2 values are the per-token probabilities released with the Podcast dataset. All six are aligned to the neural responses as the other word-level features are, by summing over words whose offset falls in $[t-0.5, t]$ s. The table reports the proportion of the unique semantic variance in IFG pars triangularis accounted for by SFM representations (Fig 5e), computed with the baseline control set and with the lexical predictors added.

Model	Layer	Baseline	+ Lexical
Whisper Large	32	44.4%	39.9%
HuBERT X-large	32	36.7%	28.2%
WavLM Large	20	38.8%	31.5%

S4 Table. Last-token versus within-step mean pooling of SFM representations. For each 250 ms step, the 12 SFM output frames (20 ms each) within the step were averaged instead of taking the final frame, with windows, context lengths, and regression settings otherwise identical. Values are shown at the layer used for each model in the main analyses: the ratio of shared to total SFM-explained neural variance (Fig 2b), and the test correlation averaged across electrodes for the SFM alone and for the joint model with interpretable features (Fig 3). Because both pooling methods were re-fitted with identical settings, the last-token values differ slightly from those in Fig 2b and Fig 3.

	Shared ratio		r, SFM alone		r, joint model	
Model (layer)	Last	Mean	Last	Mean	Last	Mean
Whisper Large (32)	0.787	0.725	0.0728	0.0760	0.0849	0.0878
HuBERT X-large (32)	0.850	0.800	0.0693	0.0721	0.0833	0.0856
WavLM Large (20)	0.809	0.755	0.0733	0.0776	0.0847	0.0869

S3 Fig. Average correlation between predicted and actual brain responses across context lengths. Average correlation between predicted and actual brain responses using embeddings from Whisper, WavLM, and HuBERT across different context lengths. The maximal correlations across layers were obtained with context lengths of 30 s for Whisper, 5 s for WavLM, and 30 s for HuBERT.

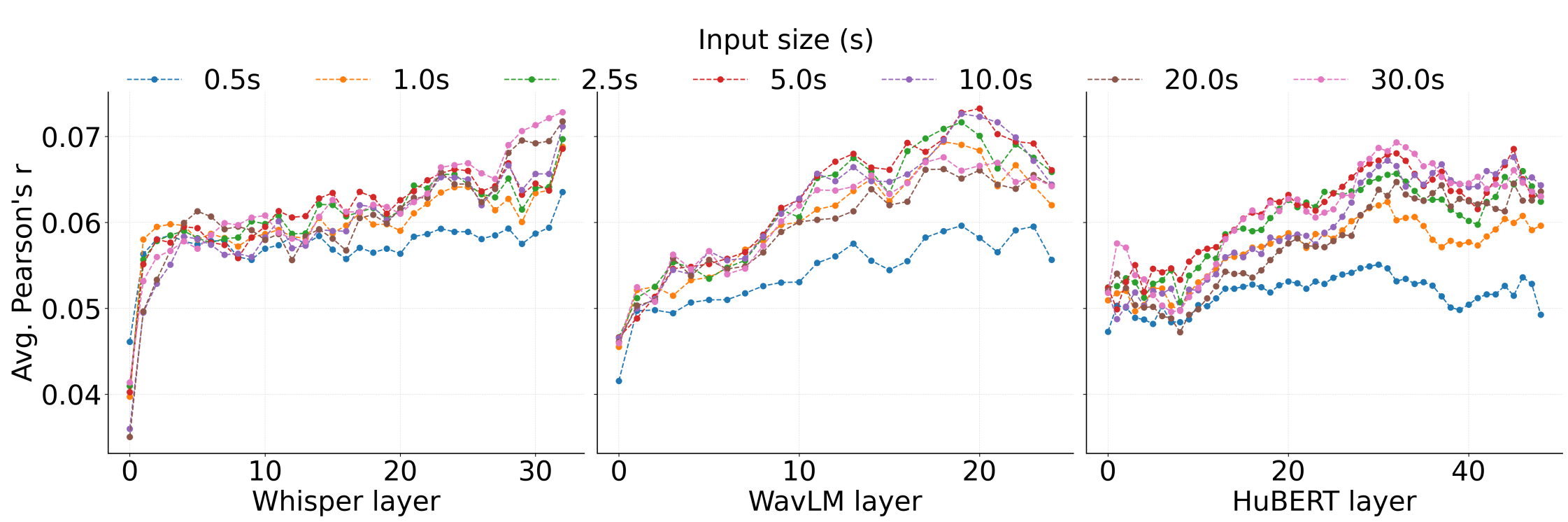

S4 Fig. Ratio of neural variance shared between interpretable feature sets and SFM representations. The ratio of neural variance shared between different interpretable feature sets and the SFM representations, relative to the total variance accounted for by each interpretable feature set.

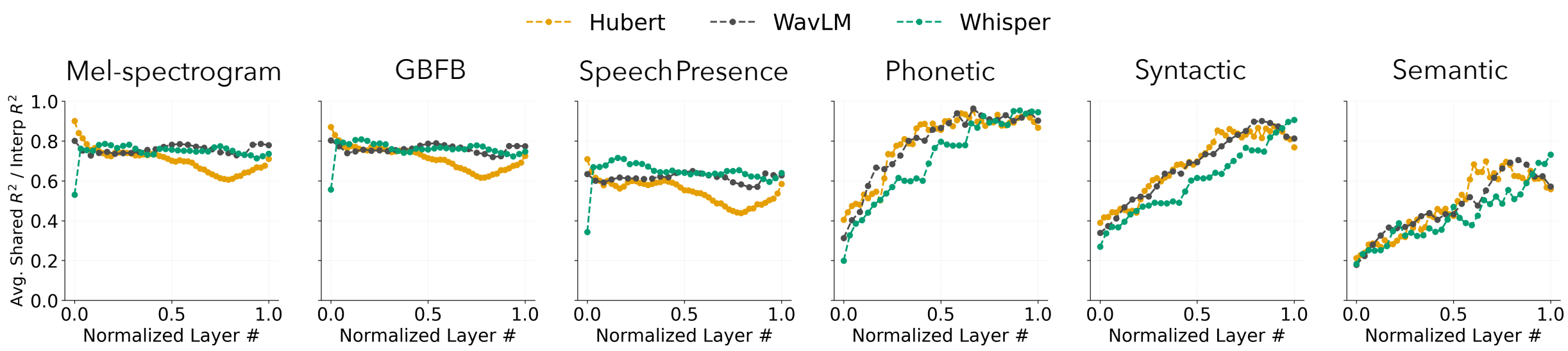

S5 Fig. Unique neural variance of WavLM and HuBERT plotted on a template brain. Unique neural variance of WavLM and HuBERT with respect to the combined set of all interpretable features, plotted on a template brain.

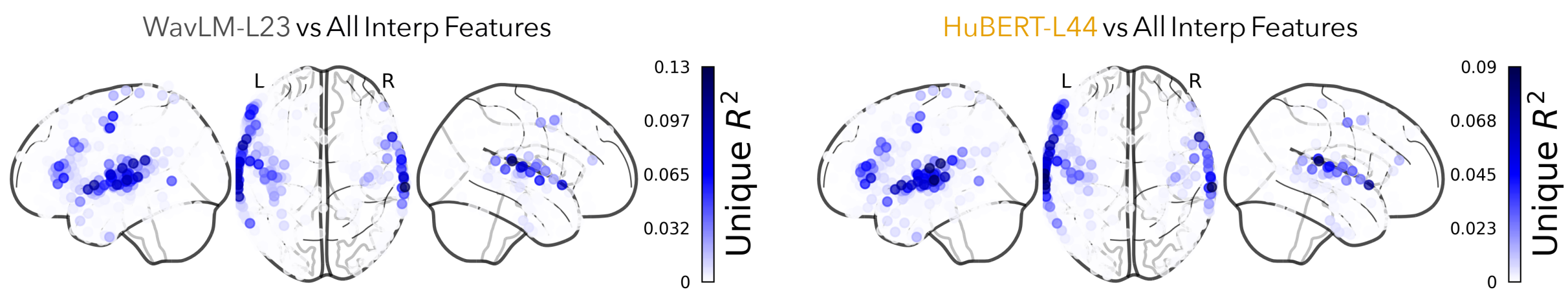

S6 Fig. Mel-spectrogram window analyses for WavLM and HuBERT. (a) Average neural variance R^2 shared between WavLM representations and mel-spectrogram segments of different temporal windows. (b) Variance R^2 of the mel-spectrogram $[t - 0.5, t]$s accounted for by the WavLM representations over different layers. (c, d) Corresponding plots for HuBERT.

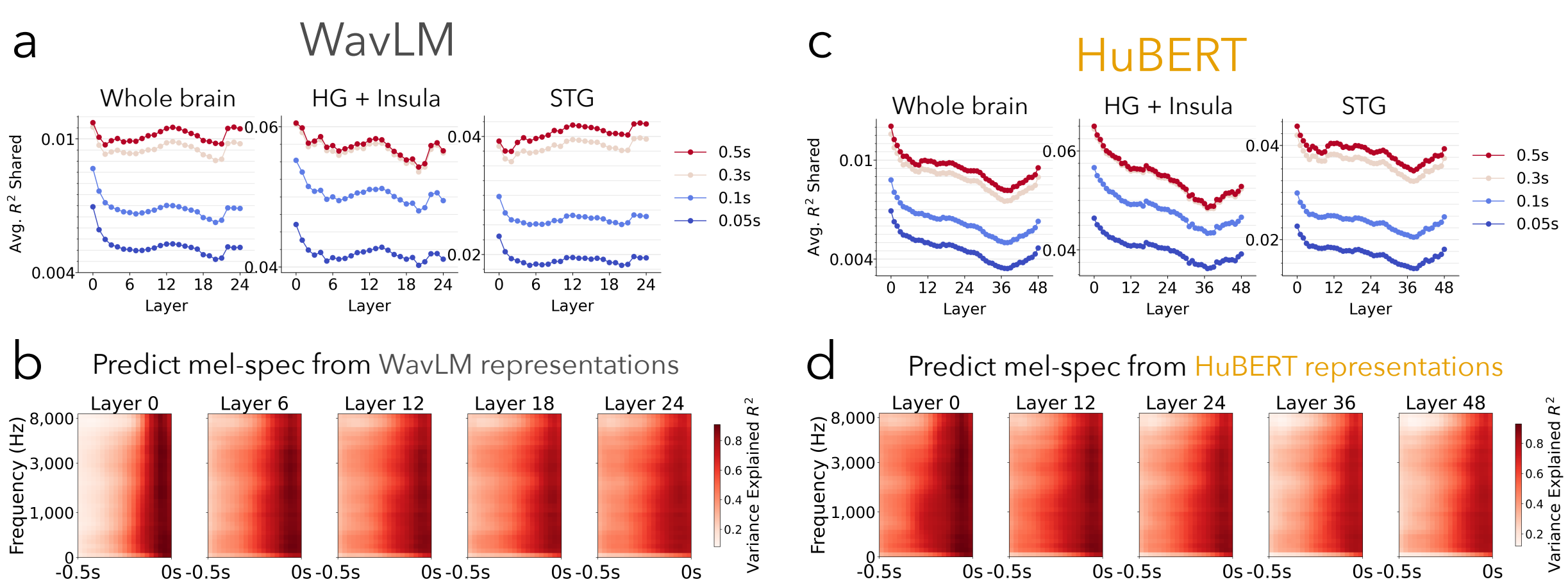

S7 Fig. Proportion of unique semantic neural variance accounted for by SFM embeddings over layers. The proportion of unique semantic neural variance accounted for by SFM embeddings over layers, averaged across electrodes in IFG-Tri.

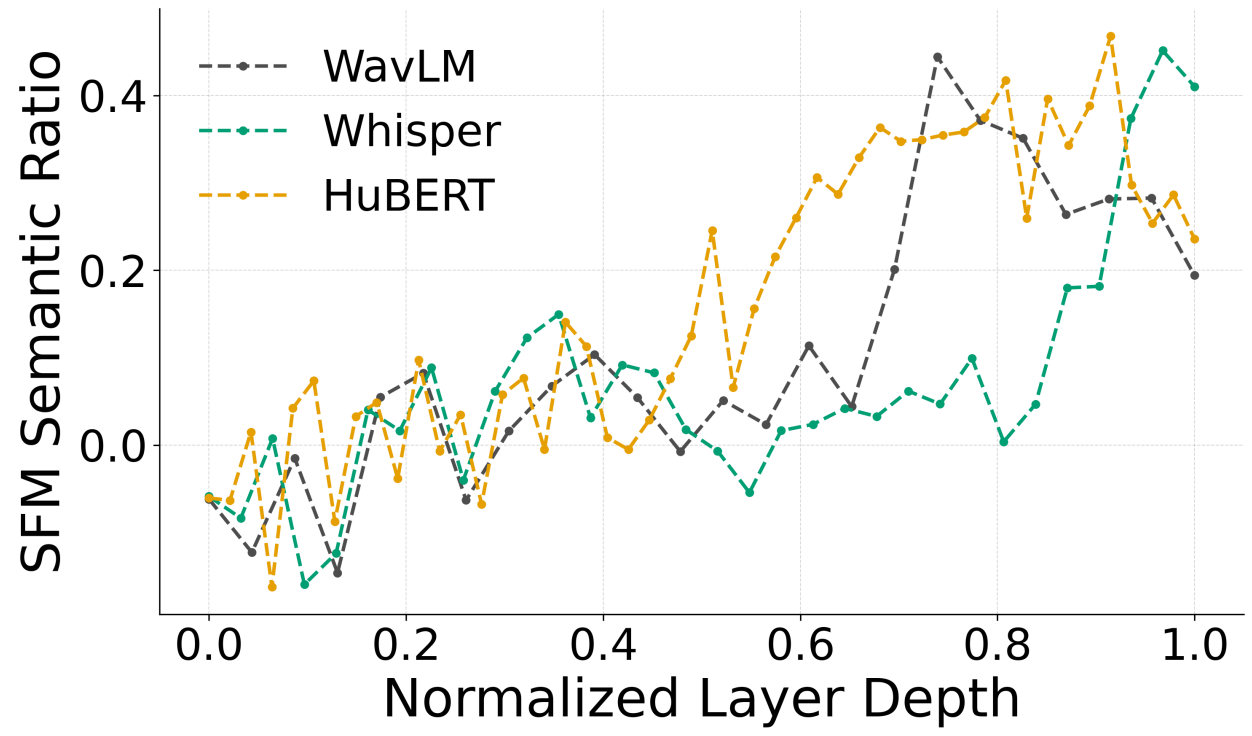

S8 Fig. Three-way variance partitioning analysis used for higher-order features. Diagram of the three-way variance partitioning analysis used to quantify the neural variance shared between higher-order features (phonetic, syntactic, and semantic) and SFM representations. Because these higher-order features also encode information about speech presence, we controlled for it explicitly within the partitioning framework.

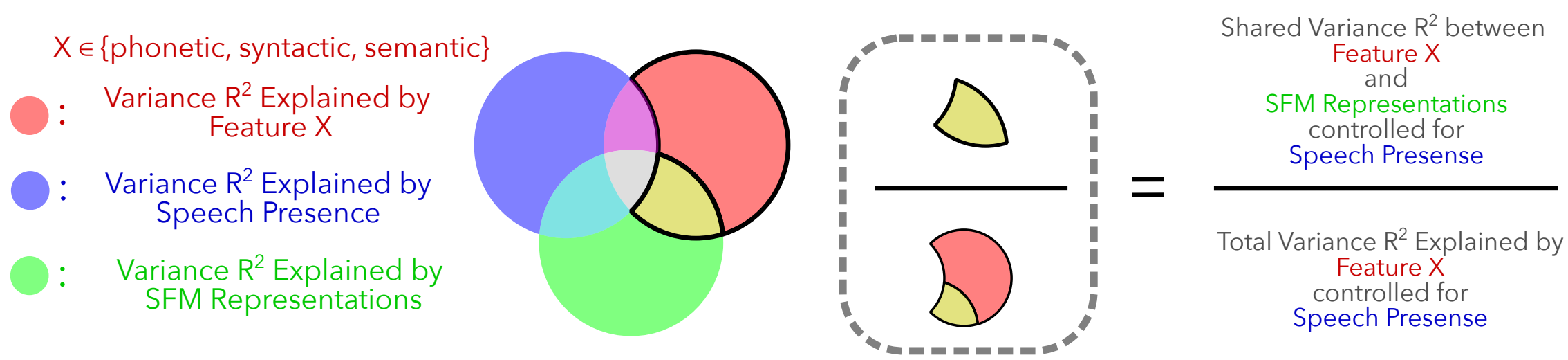

S9 Fig. Encoding performance across electrodes. Per-electrode encoding correlation r of each SFM at its best layer (Whisper-large layer 32, HuBERT-xlarge layer 32, WavLM-large layer 20), plotted on a template brain. The strongest electrodes reach $r \approx 0.7$ and are concentrated in the superior temporal gyrus and surrounding auditory cortex, whereas the mean across all 1,268 electrodes (approximately 0.07) is dominated by the many electrodes outside speech-responsive regions.

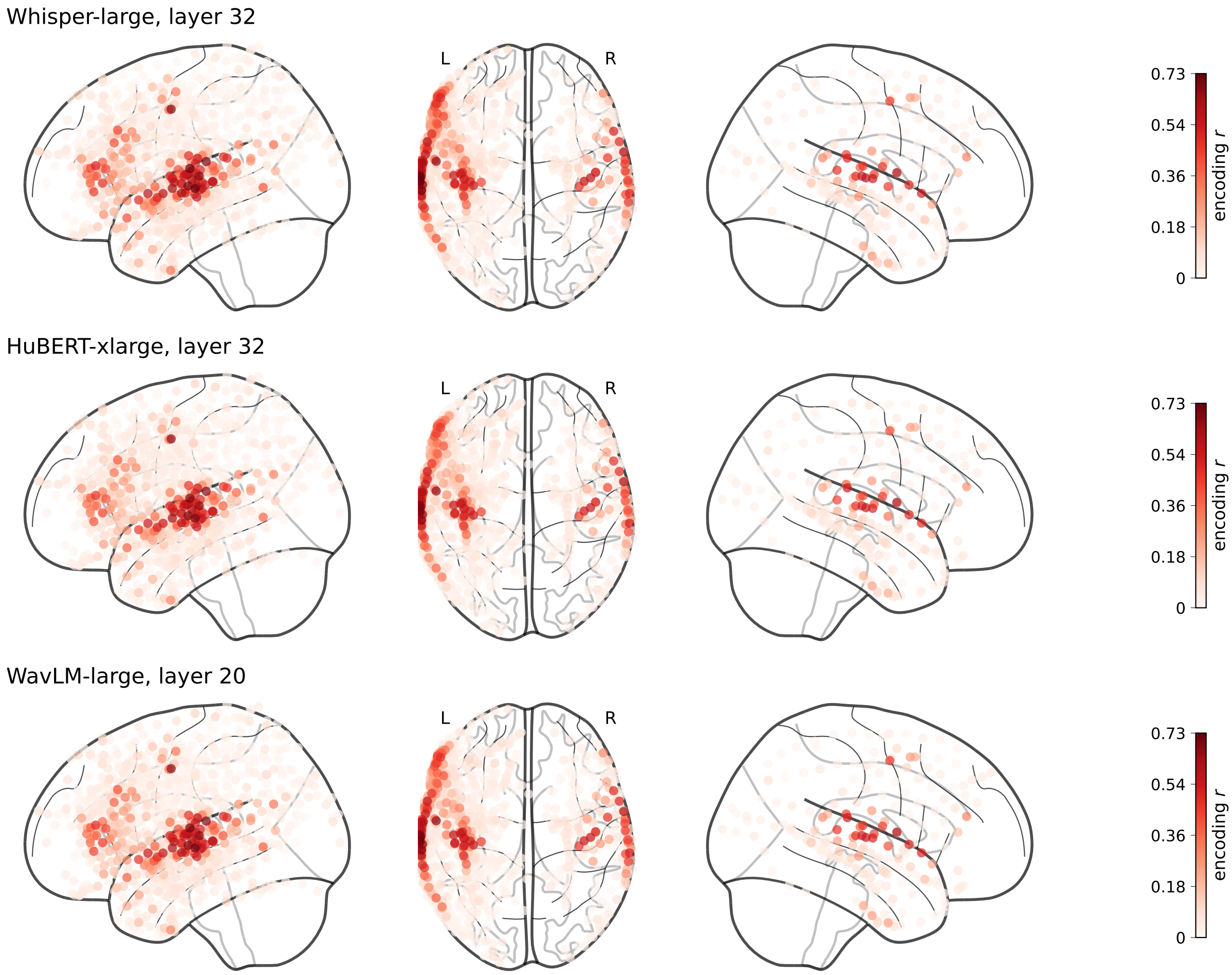

S1 Text. SFM feature extraction. Representations from speech foundation models (SFMs) were extracted using the code provided by Antonello et al. [4] (available at `https://github.com/HuthLab/encoding-model-scaling-laws`). The input audio file, totaling 1800 seconds, was divided into five contiguous 360-second segments before being fed into the SFMs. This segmentation was performed to prevent data leakage between training and test folds in the 5-fold cross-validation scheme.

S2 Text. Effect of context length on brain prediction performance. Among the context sizes tested, the context size with the highest average correlation was 30 seconds for Whisper and HuBERT, and 5 seconds for WavLM (S3 Fig). This variability highlights the need for caution when interpreting the influence of context length on encoding performance. For instance, Anderson *et al.* found that EEG encoding performance with Whisper peaked at context lengths of 5–10 seconds and interpreted this as evidence that EEG preferentially reflects the encoding of speech within that temporal window. However, it is essential to distinguish whether such patterns reflect inherent properties of neural processing or are instead artifacts of model-specific biases.

S3 Text. Variance partitioning with high-level features. In Fig 2c, the ratio for the phonetic, syntactic, and semantic features was computed using three-way variance partitioning among the SFM representations, speech probability, and the respective feature set (see S8 Fig), in order to control for speech presence information embedded within these higher-level features. For mel-spectrogram and Gabor filter bank features, no such control was applied, as they by definition represent the lowest-level confounds.
